# From Stem to Spectrum: Phytochemical Characterization of Five *Equisetum* Species and Evaluation of Their Antioxidant Potential

**DOI:** 10.3390/molecules29122821

**Published:** 2024-06-13

**Authors:** Khadijeh Nosrati Gazafroudi, Lilo K. Mailänder, Rolf Daniels, Dietmar R. Kammerer, Florian C. Stintzing

**Affiliations:** 1 Department of Analytical Development and Research, Section Phytochemical Research, Wala Heilmittel GmbH, Dorfstraße 1, DE-73087 Bad Boll/Eckwälden, Germany; khadijeh.nosrati-gazafroudi@wala.de (K.N.G.); lilo.mailaender@wala.de (L.K.M.); dietmar.kammerer@wala.de (D.R.K.); 2 Department of Pharmaceutical Technology, University of Tübingen, Auf der Morgenstelle 8, DE-72076 Tübingen, Germany; rolf.daniels@uni-tuebingen.de

**Keywords:** horsetail, phytoextract, HPLC-MS, secondary metabolites, flavonoids, DPPH assay, antioxidants

## Abstract

The Equisetaceae family, commonly known as horsetails, has been of scientific interest for decades due to its status as one of the most ancient extant vascular plant families. Notably, the corresponding species have found their place in traditional medicine, offering a wide array of applications. This study presents a comprehensive phytochemical analysis of polar secondary metabolites within the sterile stems of five distinct *Equisetum* species using HPLC–DAD-ESI-MS^n^. For this purpose, fresh plant material was extracted with acetone/water, and the resulting crude extracts were fractionated using dichloromethane, ethyl acetate, and *n*-butanol, respectively. The results reveal a complex array of compounds, including hydroxycinnamic acids, hydroxybenzoic acids, flavonoids, and other phenolic compounds. In addition, total phenolic contents (Folin–Ciocalteu assay) and antioxidant activities (DPPH assay) of the plant extracts were evaluated using spectrophotometric methods. The present comparative analysis across the five species highlights both shared and species-specific metabolites, providing valuable insights into their chemical diversity and potential pharmacological properties.

## 1. Introduction

In botanical taxonomy, horsetails belong to the genus *Equisetum* L. and are the only living members of the Equisetaceae family. This family comprises a unique group of perennial plants, characterized primarily by their mode of reproduction by spores. The precise number of extant *Equisetum* species has been a matter of ongoing scientific debate, with estimations ranging from 15 [1] to as many as 30 [2] distinct species. Their natural habitat covers a wide range of landscapes, from the northernmost reaches of Greenland and Siberia to the temperate zones of tropical America, the Cape of South Africa as well as South and South-East Asia [2]. Taxonomically, the Equisetaceae can be further divided into three monophyletic groups. Firstly, there is the subgenus *Equisetum* (e.g., *E. arvense* L., *E. telmateia* Ehrh., *E. sylvaticum* L., and *E. palustre* L.), which is perhaps the most widely known category of horsetails. Species in this subgenus are characterized by an abundance of lateral branches. This characteristic has led to the popular name ‘horsetails’. Secondly, there is the evergreen *Hippochaete* group (e.g., *E. hyemale* L.), often referred to as scouring rushes. In contrast to the subgenus *Equisetum*, the plants of the evergreen *Hippochaete* category are lacking lateral branches. Finally, the category known as *Paramochaete* represents another monophyletic group within the Equisetaceae family [2]. Each of these taxonomic groups has its own set of unique morphological characteristics. 

Remarkably, fossil records of the Equisetaceae go back to the Carboniferous period [3,4,5]. The genus has undergone minimal morphological changes over time, hence its nickname ‘living fossil’ [6,7].

Previous phytochemical investigations of the genus *Equisetum* have identified a variety of bioactive compounds. Notably, flavonoids, particularly kaempferol and quercetin derivatives, have been extensively reported in species such as *E. arvense* and *E. telmateia*. Phenolic acids, including caffeic acid and ferulic acid, are also prominent constituents [6]. Additionally, silicic acid, a distinguishing feature of *Equisetum*, has been linked to various health benefits, including bone health and diuretic effects [8]. Alkaloids, such as nicotine and palustrine, have been identified in certain species, notably *E. palustre*, and are associated with its toxicity to livestock [9].

Globally, *Equisetum* species have a long history of use in traditional medicine and are recognized in the European Pharmacopoeia [10]. Traditional applications of *Equisetum* spp. comprise the treatment of bacterial urinary tract infections, renal gravel, wound healing support, and exploit a wide range of potential health benefits including neuroprotection, hepatoprotection, anemia treatment, and antimicrobial effects [11].

*Equisetum arvense* L., commonly known as field horsetail, occupies a prominent position among all *Equisetum* species due to its traditional medicinal use. Internally, *E. arvense* has been employed for the management of urinary tract diseases, an application deeply rooted in traditional herbal practices. It has also received recognition as an effective remedy for addressing renal gravel, where it is believed to aid in the dissolution and elimination of mineral deposits within the urinary system [12]. Externally, the plant has been recommended as a valuable agent for supporting wound healing processes [12].

Other horsetail species are also used in traditional medicine all over the world, including *E. telmateia* Ehrh., which is used as an antiseptic and appreciated for its antioxidant properties, mainly attributed to polyphenols [13]. 

*E. hyemale* L. is known for its antioxidant, antifungal and antibacterial properties [14]. Recent research has even highlighted its anticancer activity on murine leukemia cells (L1210) [15].

In contrast, *Equisetum palustre* L., known as “marsh horsetail”, has been traditionally used in Turkey to treat peptic ulcers, hemorrhoids, and kidney stones [16]. However, caution should be taken due to its toxicity to livestock, linked to the presence of thiaminase and the alkaloids palustrine and nicotine [9].

Additionally, *E. sylvaticum* L. is employed as a medicinal herb to enhance blood circulation and alleviate blood stasis [17]. 

**Figure 1:**
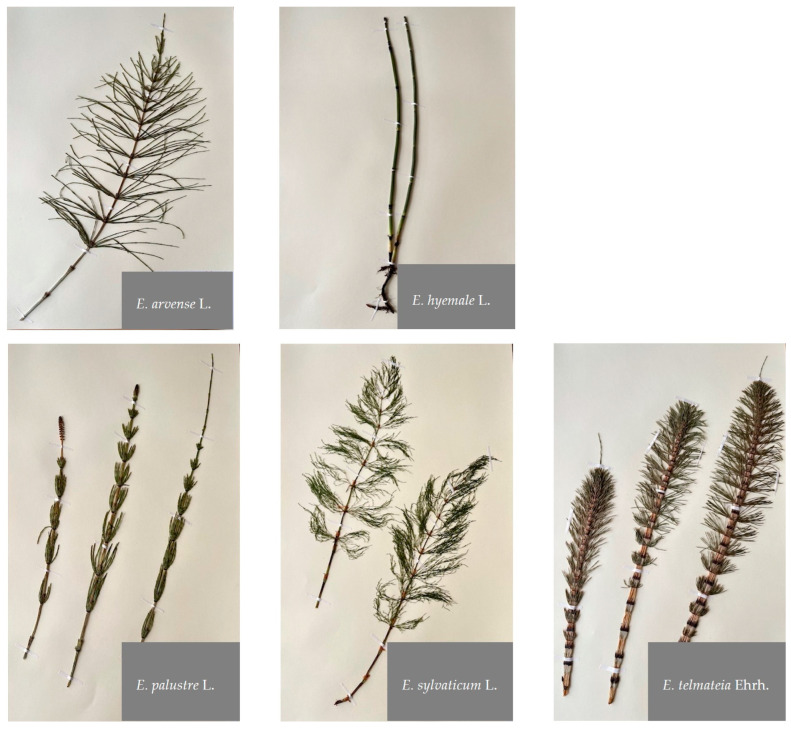
Photographic illustration of the *Equisetum* species investigated. Photos: Khadijeh Nosrati G.

The aim of the study presented here was to conduct a comprehensive analysis of polar secondary metabolites present in the sterile stems of five *Equisetum* species (Figure 1): *E. arvense* L., *E. hyemale* L., *E. palustre* L., *E. sylvaticum* L., and *E. telmateia* Ehrh. by high-performance liquid chromatography with tandem mass spectrometric detection (HPLC-MS^n^). In addition, biofunctional properties were evaluated, including semi-quantification of total phenolic contents using the Folin–Ciocalteu assay and evaluation of antioxidant capacities using the DPPH assay.

## 2. Results and Discussion

The aim of this study was to characterize and compare the polar compounds of five different *Equisetum* species, i.e., of *E. arvense* L., *E. hyemale* L., *E. palustre* L., *E. sylvaticum* L., and *E. telmateia* Ehrh., in order to identify potential chemotaxonomic markers, and to correlate their phytochemical profile with their biofunctional properties. To the best of our knowledge, there is no other work that has analyzed and compared extracts of the aforementioned *Equisetum* species on this scale.

### 2.1. Phytochemical Comparison of Five Equisetum Species

**Figure 2:**
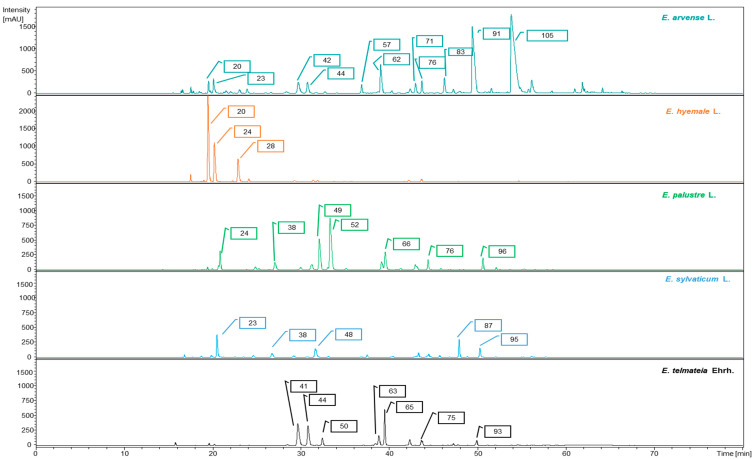
RP-HPLC-DAD chromatograms (330 nm) of *n*-butanol *Equisetum* extracts. Peak numbers refer to Table 1.

In the present work, a qualitative analysis of the chemical composition of extracts obtained from five different *Equisetum* spp. was performed using HPLC–DAD-ESI-MS^n^ operated in negative ionization mode. The UV chromatograms (UVC) of the *n*-butanol extracts from *Equisetum* spp. are illustrated in Figure 2. In total, 117 compounds (Table 1) were tentatively assigned based on their UV spectra, retention times, and MS^n^ spectra in comparison with data found in the literature. Previously identified compounds from the same botanical family were also considered in the characterization when applicable. 

Occasionally, formate adducts ([M-H+46]^−^) formed due to the presence of formic acid in the mobile phase were observed in negative ionization mode. The UV chromatograms (UVCs; Figure 2) highlight that hydroxycinnamic acids and flavonoids occurred in the different species. The phenolic subclasses comprising the majority of the 117 compounds characterized in the five different *Equisetum* species are described in the following sections. 

#### 2.1.1. Hydroxybenzoic Acids

Hydroxybenzoic acids are a significant subclass of phenolic compounds due to their diverse and vital biological functions. These compounds have remarkable antioxidant and anti-inflammatory properties. In addition, research has highlighted the beneficial effects of hydroxybenzoic acids on human health, including a reduction in the risk of cardiometabolic disorders [18].

Compound **8** exhibited a molecular ion at *m/z* 329, indicating the presence of a vanillic acid hexoside. It produced a daugther ion at *m/z* 167 due to loss of the hexosyl moiety (162 Da). Compounds **3** and **6** ([M-H]^−^ at *m/z* 315) were characterized as dihydroxybenzoic acid hexoside isomers and compound **4** ([M-H]^−^ at *m/z* 299) was assigned to a hydroxybenzoic acid hexoside. The product ions at *m/z* 153, 137 and 109 were formed by the loss of a hexosyl moiety (162 Da) and CO_2_ (44 Da) from precursor ions.

#### 2.1.2. Hydroxycinnamic Acid Derivatives

Hydroxycinnamic acids (HCA) are a group of phenolic compounds found in numerous plant species. They are known for their antioxidant properties and potential health benefits, including anti-inflammatory, anti-cancer, neuro-, and photoprotective effects. The pharmaceutical potential of hydroxycinnamic acids can be attributed to their ability to act as free radical scavengers [19]. HCAs, such as ferulic, caffeic, sinapic, and *p*-coumaric acids, have a specific chemical structure consisting of a C_6_-C_3_ phenylpropanoid backbone with a carboxyl group double-bonded to an aromatic ring, forming a conjugated π-electron system. Hydroxycinnamic acids are characterized by strong absorption in the UV region of around 220–330 nm, with variations depending on factors such as substitution patterns and functional groups present in the molecule [20]. In this study, 39 hydroxycinnamic acid derivatives and isomers were detected and tentatively characterized.

As illustrated in Table 1, compound **16** showed a molecular ion at *m/z* 311. It produced an MS^2^ base peak at *m/z* 149 ([tartaric acid-H]^−^) by the loss of a caffeoyl residue and secondary peaks at *m/z* 179 ([caffeic acid-H]^−^) and 135 ([caffeic acid-CO_2_-H]^−^). Compound **16** was therefore classified as caffeoyltartaric acid (caftaric acid) based on the above findings. Caftaric acid was only detected in the *E. sylvaticum* extract.

Compound **22** showed characteristic absorbance maxima at approx. 220 and 328 nm and exhibited a molecular ion at *m/z* 623. 

The MS^2^ base peak at *m/z* 311 ([caftaric acid-H]^−^) could be attributed to the loss of a caffeoyl tartaric acid residue. The MS^3^ base peak at *m/z* at 149 ([tartaric acid-H]^−^) was formed by the loss of a caffeoyl moiety and secondary signals at *m/z* 179 ([caffeic acid-H]^−^) and 135 ([caffeic acid-CO_2_-H]^−^). Thus, compound **22** was assigned to a dimer of caffeoyltartaric acid (caftaric acid dimer) based on the arguments presented above. This compound was detected only in *E. sylvaticum* and *E. arvense* extracts. However, it should be noted that this dimer is an artifact that is formed in the ESI source.

One metabolite (compound **58**) with a pseudomolecular ion at *m/z* 504 and an absorbance maximum at 308 nm was assigned to a caffeoyl derivative. It showed distinct fragment ions at *m/z* 341, which may point to a caffeic acid hexoside ([caffeic acid hexoside-H]^−^) due to the loss of a hexosyl moiety (162 Da), and at *m/z* 179 ([caffeic acid-H]^−^) in the MS^2^ experiment. The MS^3^ experiment generated a base peak at *m/z* 135 ([caffeic acid-CO_2_-H]^−^). This compound was detected only in *E. hyemale* extracts.

Two peaks (compounds **104** and **106**, t_R_ ~54–56 min) in the base peak chromatogram of *E. arvense* showed molecular ions at *m/z* 473. Both compounds exhibited identical MS^n^ spectra with MS^2^ base peaks at *m/z* 311 and at *m/z* 179 in the MS^3^ experiment ([caffeic acid-H]^−^) accompanied by ions at *m/z* 149 ([tartaric acid-H]^−^) and 135 ([caffeic acid-CO_2_-H]^−^). Moreover, both compounds showed characteristic absorption maxima at approximately 240 and 330 nm [20]. Based on these findings, compounds **104** and **106** were assigned to dicaffeoyltartaric acid isomers (chicoric acid). These components were detected only in *E. arvense*. 

HPLC-DAD-ESI-MS^n^ analyses indicated the presence of ferulic acid compounds ester-linked to pentose or hexose saccharides (t_R_~19.5–38.9 min) in all species investigated. Thus, as reported in Table 1, two feruloyl-pentose isomers (**39**, **47**), three feruloyl-hexose isomers (**20**, **28**, **31**), two feruloyl-hexose dimers (**19**, **27**) and one acetylated feruloyl-hexose (**60**) were assigned. A signal at *m/z* 193 corresponded to [ferulic acid-H]^−^, *m/z* 134 corresponded to ([ferulic acid-CO_2_-CH_3_-H]^−^) and secondary signals at *m/z* 149 to ([ferulic acid-CO_2_-H]^−^) and at *m/z* 178 to ([ferulic acid-CH_3_-H]^−^). 

#### 2.1.3. Flavonoids

Flavonoids are a diverse group of secondary metabolites found abundantly in plants. They play essential roles, including UV protection and defense against pathogens. Polyphenolic compounds characterized by a C_15_-carbon skeleton are known for their antioxidant properties [21]. Flavonoids show two main bands in their absorption spectra: band I (300–400 nm) and band II (240–280 nm). Band I is attributed to the electron transition of the cinnamoyl group, while Band II is attributed to the electron transition of the benzoyl group [22]. Nevertheless, UV spectroscopy has its limitations. At low analyte concentrations, UV spectra tend to exhibit irregularities and hamper compound assignment (see Table 1; Footnote f). 

The most prominent class of secondary metabolites characterized in our study were polyphenolic glycosides. 

The flavonoids characterized in the present study mainly belonged to the flavonol subclass. These include derivatives of myricetin, quercetin and kaempferol both as aglycones and in glycosylated form. The mass difference between the *m/z* values of the precursor and product ions can be used to determine the type of glycosidic substitution. Thus, differences of 146 Da are associated with a rhamnosyl moiety, 162 Da with the loss of an *O*-glucosyl or caffeoyl residue, 248 Da with a malonyl hexoside, 308 Da with a rutinosyl moiety (glucose plus rhamnose) or with a coumaroyl hexoside, 204 Da with an acetyl hexoside and finally 324 Da with a caffeoyl hexoside or a di-hexoside.

Quercetin derivatives represent the predominant flavonols in our study. The absence of quercetin in *E. telmateia* is particularly noteworthy, a pattern consistent with data previously reported in the literature [6]. 

The fragmentation pattern of compound **18** at t_R_ 19.5 min in the base peak chromatogram of *E. palustre* and compound **33** at t_R_ 24.1 min in the base peak chromatogram of *E. hyemale* ([M-H]^−^ at *m/z* 787 Da) indicated the presence of 3 hexoses linked to the aglycone by two or three *O*-glycosidic linkages.

First, the [M-H]^−^ ion at *m/z* 787 eliminated an isolated hexoside moiety (162 Da), followed by a di-hexoside unit loss (324 Da) to yield an aglycone ion at *m/z* 300. Compounds **18** and **33** were assigned to a quercetin-tri-*O*-hexoside. To the best of our knowledge, quercetin-tri-*O*-hexosides were detected for the first time in *E. palustre* and *E. hyemale*.

**Figure 3:**
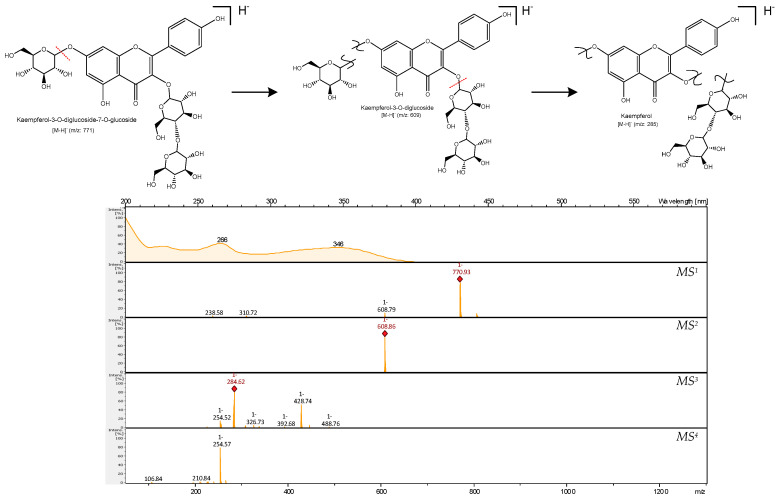
Principal fragmentation pathway of compound **24**, kaempferol-3-O-diglucoside-7-O-glucoside.

MS analysis of compound **24** (Figure 3) produced a pseudomolecular ion at *m/z* 771 ([M + hex + hex + hex-H]^−^), corresponding to a kaempferol tri-hexoside. It showed a distinct ion at *m/z* 609 ([kaempferol + hex + hex-H]^−^) by the loss of a hexoside moiety (162 Da) in the MS^2^ experiment. The MS^3^ generated an ion at *m/z* 429 ([kaempferol + hex-H]^−^) due to the loss of another hexoside moiety, ending with the loss of the third hexose leading to *m/z* 285 ([kaempferol-H]^−^). This kaempferol tri-hexoside was only detected in *E. palustre* extracts. Thus, the presence of quercetin and kaempferol tri-glycosides in *E. palustre* may be used as a chemotaxonomic marker to distinguish it from the other three species, i.e., *E. arvense*, *E. telmateia* and *E. sylvaticum*.

Compound **26** at t_R_ 23.0 min exhibited an *m/z* ratio of 641. A hexose moiety (162 Da) was released upon fragmentatione, followed by a second hexoside unit (162 Da) to yield the aglycone ion ([aglycone-H]^−^) at *m/z* 317. Compound **26** was, therefore, assigned to a myricetin di-hexoside for the first time in *E. palustre*. The occurrence of myricetin in *E. arvense*, as reported in the literature [6], could not be confirmed. 

Moreover, compound **26** may represent a chemotaxonomic marker for *E. palustre*, as it was not found in the other species.

Compounds **43** and **48** showed pseudomolecular ions at *m/z* 609. Their MS^2^ and MS^3^ spectra revealed ions at *m/z* 447 and 285 resulting from successive losses of 162 Da, suggesting the presence of two hexosyl moieties.

MS^4^ of the [aglycon-H]^−^ ion at *m/z* 285 yielded a signal at *m/z* 255, consistent with kaempferol. Thus, Compounds **43** and **48** were characterized as kaempferol- di-*O*-hexosides. 

Compound **76** showed an [M-H]^−^ ion at *m/z* 593. The MS^2^ experiment gave a base peak at *m/z* 285, due to the simultaneous loss of rhamnose (146 Da) and a hexose (162 Da), forming a disaccharide moiety. Further fragmentation of the ion at *m/z* 285 was identical to that of kaempferol. Compound **76** was therefore characterized as kaempferol 3-hexoside-7-rhamnoside.

MS analysis of compound **66** yielded an [aglycon-H]^−^ ion at *m/z* 301, resulting from the simultaneous loss of a rhamnose (146 Da) and hexose (162 Da) moiety. Further fragmentation of the ion at *m/z* 301 was consistent with quercetin. This compound was therefore assigned to quercetin-3-glucoside-7-rhamnoside.

In reversed phase HPLC, flavonoids with a higher degree of glycosylation show shorter retention times. In addition, acylation with hydroxycinnamic acids affects chromatographic mobility differently depending on the glycosidic substitution of the flavonoid. Glycosides typically elute first, followed by their acetate and malonate conjugates, and finally the aglycones as their hydrophobicity increases. In general, retention times follow the following sequence: triglycosides < diglycosides < monoglycosides < acetate and malonate conjugates < aglycones [23].

Based on characteristic neutral losses of 248 Da or 204 Da, corresponding to the elimination of a glycosyl-malonyl or glycosyl-acetyl moiety with the subsequent formation of a dominant product ion derived from free aglycone structures, we were able to characterize malonyl- (**57**, **98**, **103**) in *E. palustre* and *E. sylvaticum* and acetyl-conjugated (**56**, **64**, **73**, **86**, **89**, **90**, **94**, **97**, **99**, **100**) glycosides in *E. arvense*, *E. telmateia*, *E. sylvaticum*. Losses of glycosyl malonyl or glycosyl acetyl moieties resulted in [aglycone-H]^−^ ions at *m/z* 285 and 301, indicating the presence of malonylated or acetylated glycosides of kaempferol and quercetin, respectively.

*C*-glycosylated flavonoids, characterized by saccharides directly attached to the aglycone at ring A via a C-C bond, consistently have substituents at positions 6 (C-6) and/or 8 (C-8) of the aglycone moiety. In negative ionization mode, these compounds exhibit distinct fragmentation patterns, with hexose substituents undergoing cross-cleavage resulting in losses of 120 Da and 90 Da, whereas pentose substituents experience losses of 90 Da and 60 Da. 

For compound **50** (*m/z* 593 [M-H]^−^), the fragment ions at *m/z* 473 [(M-H)-120]^−^, at 383 [(M-H)-210]^−^ and 353 [(M-H)-240]^−^ in the MS^2^ spectrum were consistent with those reported by Wang et al. [24] for di-*C*-hexosyl flavones, thus suggesting the presence of vicenin 2. To the best of our knowledge, vicenin 2 was detected for the first time in *E. arvense* and may represent a chemotaxonomic marker, as this compound was not found in the other species.

#### 2.1.4. Stilbenoids

Stilbenoids belong to the group of non-flavonoid phenolic compounds. Generally, stilbenoids are considered phytoalexins. Their presence in plant tissues is associated with resistance to fungal diseases as caused e.g., by *Botrytis cinerea*, although they may also appear in response to abiotic stress such as UV irradiation [25]. 

**Figure 4:**
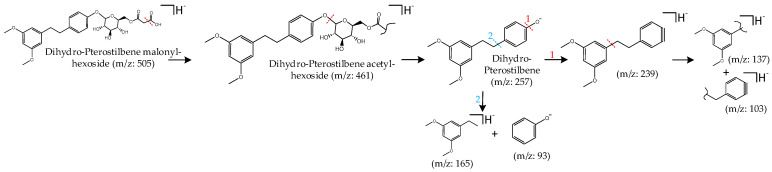
Two postulated fragmentation pathways (1 and 2) for compound **88**, dihydro-pterostilbene malonyl-hexoside.

Compound **88** generated a pseudomolecular ion at *m/z* 505 after a CO_2_ moiety loss it generated a fragment at *m/z* 461. Its MS^2^ base chromatogram showed a fragment at *m/z* 257 after the release of an acetyl hexoside moiety (204 Da). In negative ionization mode, further fragments at *m/z* 239 ([M-H_2_O]^−^) and *m/z* 137 ([M-H-C_8_H_10_O_2_]^−^) were detected by the breakage of the C2-chain. The fragments ions at *m/z* 165 and *m/z* 93 were formed in the same fragmentation pattern. Based on a comparison with data reported by Wang et al. [26] compound **88** was characterized as dihydro-pterostilbene malonyl-hexoside. Furthermore, compounds **111** and **113** were tentatively assigned as lunularic acid derivatives. To confirm these results, further investigations are required. To the best of our knowledge, this is the first time that stilbenes have been detected in any *Equisetum* species. The fragmentation behavior of compound **88** is shown in Figure 4. 

#### 2.1.5. Further Minor Compounds

Moreover, our investigations revealed further substance classes, including benzophenone derivatives like maclurin (compounds **52**, **55** and **67**) and the chalone derivative phloridzin (compounds **62** and **74**). Additionally, derivatives of formononetin (isoflavone) and biogenic amines such as compounds **9** and **10** were detected.

#### 2.1.6. Phytochemical Comparison of the Equisetum Species

The present study highlights the complex and diverse chemical composition of the different *Equisetum* species. Notably, several phenolics were characterized, including hydroxybenzoic and hydroxycinnamic acid derivatives (e.g., vanillic acid hexoside, caftaric cid, dicaffeoyltartaric acid, caffeoyl and feruloyl derivatives), chalcones such as phloridzin, flavonoids and stilbenoids (e.g., pterostilbene). The subgroup of flavonols, including myricetin, quercetin, and kaempferol, emerged as the predominant subgroup. Our analyses revealed distinct chemical composition patterns and differences between the species. Table 2 presents a selection of chemotaxonomic markers to distinguish *Equisetum* species. 

The absence of caffeic acid derivatives in the phytochemical profile of *E. palustre* is noteworthy, whereas they were present in the other species. This might be due to concentration effects as traces of caffeic acid derivatives were detectable in the base peak chromatogram of *E. palustre*. In contrast, flavonoid tri-glycosides were only present in *E. palustre* and *E. hyemale*, whereas a quercetin tri-*O*-hexoside was detected for the first time in these two species. 

Flavonoid acetyl-glycosides were ubiquitous in all species. However, quercetin and Dicaffeoyltartaric acid (chicoric acid) were the main constituents of *Equisetum arvense*. Vicenin 2 was detected for the first time in *E. arvense* and may represent a chemotaxonomic marker, as this compound was not found in the other species. Conversely, myricetin di-hexoside was only found in *E. palustre*. These characteristics might be used to differentiate between the species, e.g., to distinguish the toxic *E. palustre* from *E. arvense*. 

*E. hyemale* differs morphologically and chemically from the other species, with ferulic acid derivatives being the main constituents. Other hydroxycinnamic acids as well as flavonoids could only be detected in trace amounts. 

Furthermore, our study revealed a new group of compounds, stilbene derivatives, previously unknown in *Equisetum* species, which were detected in *E. telmateia* and *E. sylvaticum*. In conclusion, this study offers valuable insights into the phytochemical differences of closely related *Equisetum* species and proposes some compounds that may be used as taxonomic markers to distinguish these species. However, further studies are needed to validate and extend these results.

**Table 1:** Physicochemical characteristics, occurrence, and peak assignment of metabolites detected in *n*-butanol extracts of *Equisetum arvense* (EA), *Equisetum telmateia* (ET), *Equisetum palustre* (EP), *Equisetum sylvaticum* (ES) and *Equisetum hyemale* (EH) using HPLC-DAD-ESI-MS^n^ (negative ionization mode).

Compound No.	t_R_ [min]	Peak Assignment	Compound Class	λ_max_ [nm]	Mass Spectrometric Data [*m/z*]/ [M-H]^−^				Reference	Detection				
					MS^1^ (Pseudomolecular Ion/Species)	MS²	MS³	MS^4^		EA	ET	EP	ES	EH
1	2.0	Sucrose dimer	Others	ND ^[a]^	683	341	179, 142, 113		[27]	×	×	√	√	√
2	13.1	*p*-Hydroxybenzoic acid-*O*-hexoside Dimer	HBA	206, 360	599	299	137		[28]	×	√	×	×	×
3	13.3	Dihydroxybenzoic acid hexoside isomer	HBA	206, 362	315	153	109		[28]	×	×	×	×	√
4	13.4	*p*-Hydroxybenzoic acid *O*-hexoside	HBA	252	299	137			[28]	×	×	√	×	×
5	13.7	Palustrine ^[c]^	Others	360, 380	310	157			[9]	×	×	√	×	×
6	14.0	Dihydroxybenzoic acid hexoside isomer	HBA	206, 316, 360	315	153	109		[28]	×	×	√	√	×
7	14.1	3′,4′-Dihydroxypropiophenone-3-*O*-glucoside	Others	360, 388	327	165, 137			t. a.	×	√	×	×	×
8	15.5	Vanillic acid hexose isomer	HBA	206, 228, 282	329	167, 151			[29]	×	×	×	√	√
9	15.8	Caffeoylputrescine	Others	238, 318	249	207, 178, 135			t. a.	×	√	×	×	×
10	16.1	Acetylspermidine	Others	390	188	146	118		t. a. [9]	×	×	√	×	×
11	16.2	Hydroxyphenylethyl-coumaroyl-hexoside	HCA	ND ^[a]^	445	137, 179			t. a.	×	√	×	×	×
12	17.6	Caffeoyl hexose	HCA	294, 310, 316	341	179	135		[29]	√	×	√	×	√
13	17.7	Dihydrochalcone *C*-hexoside	Others	ND ^[a]^	329	209	167, 125, 191		t. a.	×	√	×	×	×
14	17.8	Vanillic acid hexose isomer	HBA	206, 228, 282	329	167			[29]	×	×	×	√	×
15	18.1	Caffeoyl-coumaroyl-hexoside	HCA	382	487	341, 179, 135	159		t. a.	×	√	×	×	×
16	18.8	Caftaric acid	HCA	206	311	149, 179, 135			[30] [31]	×	×	×	√	×
17	19.3	Hydrocaffeic acid hexoside	HCA	202, 384	343	181	137		[29]	×	√	×	√	×
18	19.5	Quercetin-tri-*O*-hexoside isomer	FA	274, 326 ^[f]^	787	625	300	255	[32] [23]	×	×	√	×	×
19	19.5	Feruloyl hexose Dimer	HCA	216, 232, 288, 308	711	355	193		[29]	√	×	×	×	√
20	19.7	Feruloyl hexose isomer	HCA	316	401 ^[d]^	355	193	149	t. a.	×	√	×	×	×
21	19.8	Caffeoyl hexose isomer	HCA	230, 292	341	179	135		[29]	√	×	×	×	×
22	20.2	Caftaric acid dimer	HCA	220, 328	623	311	179, 149, 135		[30] [31]	√	×	×	√	×
23	20.5	Hydro-Ferulic acid-hexoside	HCA	266, 320, 324	357	195	136		[29]	×	√	×	×	√
24	20.9	Kaempferol 3-diglucoside-7-glucoside	FA	266, 346	771	609	284, 429	255	[23]	×	×	√	×	×
25	21.7	18-Desoxypalustrine ^[c]^	Others	ND ^[a]^	294	157, 251			t. a. [9]	×	×	√	×	×
26	23.0	Myricetin dihexoside	FA	360	641	479	317	271, 244	t. a. [33]	×	×	√	×	×
27	23.0	Feruloyl hexose dimer	HCA	292	711	355	193, 149		t. a. [29]	×	×	×	×	√
28	23.1	Feruloyl hexose isomer	HCA	304	355	193	149, 178		[29]	√	×	×	×	×
29	23.2	Methyl-phloretic acid glucoside	HCA	200, 280	343	181	166		t. a.	×	×	×	√	×
30	23.4	Caffeoyl hexose isomer	HCA	382	341	179			[29]	×	√	×	×	×
31	23.5	Feruloyl hexose isomer	HCA	204, 310	355	193	178, 134, 149		[29]	×	×	√	√	×
32	23.9	Quercetin diglucoside isomer	FA	256, 352	625	463	301	271	[29]	√	×	×	×	×
33	24.1	Quercetin-tri-*O*-hexoside isomer	FA	270, 352	787	625	300	255, 271	[32]	×	×	×	×	√
34	24.6	Coutaric acid	HCA	206, 228, 310	295	149, 163	131		[30]	×	×	√	√	×
35	25.3	Quercetin-di-*O*-hexoside isomer	FA	258, 352	625	463	301, 343	271, 255	[34]	×	×	√	×	×
36	26.5	Quercetin-*p*-coumaroyl-di-hexoside	FA	266, 358	771	609	301, 271	256, 151	^[b]^ t. a.	×	×	√	×	×
37	26.8	*p*-Coumaroyl-pentose	HCA	204, 310	295	163	119		[35]	×	×	√	√	×
38	29.0	1-*O*-Sinapoyl-glucoside isomer	HCA	230, 284, 360, 380	433	387	163		[30] [36]	×	√	×	×	×
39	29.2	Feruloyl-pentose isomer	HCA	322	325	193	134		[35]	×	×	×	√	×
40	29.7	Caffeoylmalic acid dimer isomere	HCA	218, 326	591	295	179		[37]	×	√	×	×	×
41	29.8	Caffeoylmalic acid	HCA	220, 326	295	179, 133,115			[30]	√	×	×	×	×
42	30.0	Methyl-kaempferol dihexoside isomer	FA	230, 274 ^[f]^	625	463	300	255, 271	[30] [38]	×	×	√	×	×
43	30.8	Kaempferol-*O*-dihexoside isomer	FA	264, 342	609	447	284	255	[30]	√	√	×	×	×
44	31.2	Caffeoylmalic acid dimer isomere	HCA	242, 328	591	295	179, 133		[30]	×	×	√	×	×
45	31.4	Methyl-kaempferol dihexoside isomer	FA	280 ^[f]^	419	299	255		[30] [38]	×	√	×	×	×
46	31.5	Feruloyl-sulfonyl-malate-hexoside	HCA	234, 324	551	389, 193	134, 149		t. a.	×	×	×	×	√
47	31.7	Feruloyl-pentose isomer	HCA	326	325	193	134		[35]	×	×	×	√	×
48	32.1	Kaempferol-*O*-dihexoside isomer	FA	264 ^[f]^	609	447	284	255	[23]	×	×	√	×	×
49	32.5	Kaempferol-coumaroyl diglucoside	FA	266, 346	755	593	285	257	[29]	×	√	×	×	×
50	32.8	Vicenin 2	FA	328 ^[f]^	593	473, 383, 353	297, 191		[24]	√	×	×	×	×
51	33.4	Kaempferol-coumaroyl diglucoside isomer	FA	266, 346	755	593	285	257	[29]	×	×	√	×	×
52	33.8	Maclurin-*O*-hexoside isomer	Others	260, 374	423	261, 287	217		t. a.	×	×	×	×	√
53	34.2	1-*O*-Sinapoyl-glucoside isomer	HCA	ND ^[a]^	431 ^[d]^	385	205, 179		[30]	×	×	×	×	√
54	35.0	Feruloyl-sulfonyl-malate-hexoside isomer	HCA	310	551	389	193		t. a.	×	×	×	×	√
55	35.2	Maclurin-*O*-hexoside isomer	Others	228, 370	423	261	217		t. a.	×	×	√	×	×
56	36.9	Quercetin-acetyl-di-hexoside	FA	266 ^[f]^	651	489	285	255	t. a.	√	×	×	×	×
57	37.5	Kaempferol-3-*O*-6″-malonyl-diglucoside	FA	266, 344	695	651	489	284	[39]	×	×	×	√	×
58	38.1	Caffeoyl derivative	HCA	308	504	179, 342	135		[40]	×	×	×	×	√
59	38.3	Hydrocaffeic acid-acetyl-hexoside	HCA	ND ^[a]^	385	325	181	166	t. a.	×	×	×	√	×
60	38.9	Ferulic acid (6-acetyl-hexoside)	HCA	308	397	193	149, 134		t. a. [29]	×	×	×	×	√
61	39.1	Quercetin-diglucoside isomer	FA	352	625	505	343, 300	271	[29]	√	×	×	×	×
62	39.2	Phloridzin	Others	ND	435	273	255	107, 149	[41]	×	√	×	√	×
63	39.2	Malic acid *p*-coumarate	HCA	308	279	163, 133, 119			[42]	×	×	√	×	×
64	39.6	Kaempferol-acetyl-diglucoside	FA	268, 346	651	489, 285	255		[29]	×	√	×	×	×
65	39.6	*p*-Coumaric acid	HCA	228, 312	163	119			[36]	×	×	√	×	×
66	40.2	Quercetin-3-glucoside-7-rhamnoside	FA	204 ^[f]^	609	447, 301	271, 151		[43]	×	×	×	√	×
67	40.9	Maclurin-malonyl-hexoside	Others	228, 274, 360	423, 508	287, 261	99, 153		t. a.	×	×	√	×	×
68	41.4	Quercetin-(caffeoyl)-glucoside	FA	230, 370	625	301	151, 178.44		[32]	×	×	√	×	×
69	42.3	Ferulic acid	HCA	326	193	134, 178			[36]	√	×	×	×	√
70	43.0	Genkwanin-6-*C*-hexoside	FA	232, 250, 298 ^[f]^	509	463, 283	268		t. a.	√	×	×	×	×
71	43.0	Myricetin-glucoside	FA	260, 382	479	317	299	271(M-H)^2-^	[33]	×	×	√	×	×
72	43.1	Caffeic acid/cinnamic acid dimer	HCA	204, 314	455	309	112, 19		t. a.	×	×	×	√	×
73	43.4	Quercetin 3-*O*-(4″-*O*-acetyl) rutinoside	FA	356	695 ^[e]^	651	505, 301	271	[44]	×	×	×	√	×
74	43.6	4-Deoxyphloridzin	Others	204, 268, 346	465, 419	257	239	195	[41]	×	√	×	×	×
75	43.7	Kaempferol-caffeoyl-hexoside	FA	266, 346sh	609	285, 429	255		t. a.	√	×	√	×	√
76	44.4	Kaempferol-3-hexoside-7-rhamnoside	FA	266, 346	593	447, 285	284	255	[43]	×	×	×	√	×
77	44.7	unknown	-	310	429	215, 149			-	×	×	×	×	√
78	45.2	Di-caffeoyl-cinnamic acid	HCA	234, 282sh	429, 489	265, 309	147		t. a.	×	×	×	√	×
79	45.5	Caffeoyl hexose isomer	HCA	310	342	180, 222,252, 282	207, 135		[29]	√	×	×	×	×
80	45.5	Apigenin 6-*C*-hexoside	FA	ND ^[a] [f]^	449	269	207, 251		t. a.	×	×	×	×	√
81	45.8	Quercetin-3-*O*-rutinoside (rutin)	FA	258, 356	609	301	271, 178, 255		[29] [34]	×	×	√	√	×
82	46.3	Quercetin-*O*-hexoside	FA	204, 256sh, 352sh	463	301	178, 271, 255, 151		[45]	√	×	×	×	×
83	46.9	Hyperoside (Quercetin 3-*O*-galactoside)	FA	204, 228, 278sh ^[f]^	463	301	271, 151, 178 228		[30]	×	×	×	√	×
84	47.0	2″-*O*-Galloylvitexin	FA	280 ^[f]^	583, 415	313	269		t. a.	×	√	×	×	×
85	47.3	Ononin	FA	322 ^[f]^	429	267	223	145	t. a.	√	×	×	×	×
86	47.9	Kaempferol-3-acetyl-glucoside-7-rhamnoside isomere	FA	266, 346	679 ^[e]^	635	489	285	t. a. [43]	×	×	×	√	×
87	48.1	Apigenin-7-*O*-glucoside	FA	330 ^[f]^	431	269	183, 149		[46]	√	×	×	×	×
88	48.9	Dihydro-Pterostilbene-(6-malonyl-hexoside)	Stilbenoid	280	505, 461	257	239,165,137,93		t. a. [26]	×	√	×	×	×
89	48.9	Kaempferol-3-acetyl-glucoside-7-rhamnoside isomere	FA	204, 230, 278 ^[f]^	635	489	284	255	t. a. [43]	×	×	×	√	×
90	49.5	Quercetin 3-(6″-acetylglucoside)	FA	210, 256, 352	505	301	255, 178, 151		[45]	√	×	×	×	×
91	49.6	Alkaloid	-	ND ^[a]^	473	160	112		-	×	×	×	×	√
92	50.0	Kaempferol-coumaroyl glucoside	FA	280 ^[f]^	593	285	257	226	[29]	×	√	×	×	×
93	50.0	Quercetin-*O*-hexoside isomer	FA	236, 276sh ^[f]^	463	301	255, 271		[34]	×	×	√	×	×
94	50.4	Quercetin-3-acetyl-glucoside isomer	FA	204, 356	505	301	271, 255		[45]	×	×	×	√	×
95	50.7	Kaempferol-coumaroyl glucoside	FA	266, 346sh	593	285	255, 229, 178		[29]	×	×	√	×	×
96	51.2	Kaempferol glucoside	FA	280 ^[f]^	447	285	255		[29]	×	√	×	×	×
97	51.6	Quercetin 3-(6″-acetylglucoside) isomer	FA	204, 256, 352	505	445, 301	271, 255, 151		[45]	√	×	×	×	×
98	52.1	Quercetagetin-malonyl-hexoside	FA	260, 384	565	521	317	299, 271, 255	t. a.	×	×	√	×	×
99	52.1	Quercetin-3-acetyl-glucoside isomer	FA	232, 274sh, 360	505	301	271		[45]	×	×	×	√	×
100	52.2	Methyl-Kaempferol-acetyl-glucoside	FA	342	505	300	271, 255, 151		[30] [38]	√	×	×	×	×
101	52.7	Schaftoside/ Isoschaftoside isomer (Apigenin-glucoside-arabinoside)	FA	280 ^[f]^	563	503	341	311	[24]	×	√	×	×	×
102	53.4	Schaftoside/Isoschaftoside isomer (Apigenin-glucoside-arabinoside)	FA	280 ^[f]^	563	503	341	311	[24]	×	√	×	×	×
103	53.7	Quercetin-malonyl-hexoside	FA	256, 370	505, 549	301	151, 178, 205, 255		(b) ^[b]^ t. a.	×	×	√	×	×
104	54.3	Dicaffeoyltartaric acid isomer	HCA	244, 220, 328	473	311	179, 149, 135	87	[47] [30]	√	×	×	×	×
105	55.1	Flavonol *C*-hexoside isomer	FA	ND ^[a] [f]^	431	251	207, 163		-	×	×	×	×	√
106	56.2	Dicaffeoyltartaric acid isomer	HCA	238, 324	473	311	179, 149, 135		[47] [30]	√	×	×	×	×
107	56.5	*N*-Formylpalustrine	Others	336	635	468	244, 338	161, 201	[9]	×	×	√	×	×
108	57.0	Flavonol *C*-hexoside isomer	FA	ND ^[a] [f]^	431	251	207, 163		-	×	×	×	×	√
109	57.9	*N*-Formylpalustrine isomer	Others	336	635	468	244, 338	227, 202	[9]	×	×	√	×	×
110	58.5	Formononetin-acetyl-hexoside	FA	312 ^[f]^	471, 413	267	223		t. a.	√	×	×	×	×
111	59.2	Lunularic acid-hexoside	Stilbenoid	236	419	257	213		t. a.	×	×	×	√	×
112	61.1	Formononetin-malonyl-hexoside	FA	322 ^[f]^	515	267	161		t. a.	√	×	×	×	×
113	63.6	Lunularic acid- malonyl-hexoside	Stilbenoid	238	505	461	213		t. a.	×	×	×	√	×
114	64.8	Caffeic acid derivative	HCA	ND ^[a]^	457	179	119		t. a.	×	×	×	×	√
115	66.0	1,3-Dihydroxyanthraquinones acetyl-hexoside	Others	224	443	401	239		t. a. [48]	×	√	×	√	×
116	66.8	Sinapoyl malate-hexosyl-pentoside	HCA	230, 280sh	635	501, 339	324	309	t. a.	×	√	×	×	×
117	68.1	Dicaffeoyl-quinic acid (Cynarin)	HCA	234	515	335	291, 179		t. a.	×	√	×	×	×

^[a]^ ND—not detected. ^[b]^ Characterized based on MS^n^ data and public database of FooDB. ^[c]^ Alkaloid detected in positive ionization mode. ^[d]^ Formate adduct ([M-H + HCOOH]^−^). ^[e]^ Carbon dioxide adduct ([M-H + CO_2_]^−^). ^[f]^ Due to very low quantitative amounts, the typical absorption maxima at 340–370 nm is missing. HBA: Hydroxybenzoic acid derivative. HCA: Hydroxycinnamic acid derivative. FA: Flavonoid derivative. √: Present. ×: Not present. t. a.: tentatively assigned.

**Table 2:** Selected species-specific chemotaxonomic markers.

*E. arvense*	*E. hyemale*	*E. palustre*	*E. sylvaticum*	*E. telmateia*
Flavonoid acetyl-glycosides (quercetin derivative	Flavonoid tri-glycosides (e.g., quercetin tri-*O*-hexoside *)	Flavonoid tri-glycosides (e.g., quercetin tri-*O*-hexoside *)	Flavonoid acetyl-glycosides (kaempferol derivative)	Flavonoid acetyl-glycosides (kaempferol derivative)
Dicaffeoyltartaric acid	Ferulic acid derivatives	Myricetin di-hexoside *	Stilbenoid * (e.g., lunularic acid derivatives)	Stilbenoid * (e.g., pterostilbene derivative)
Vicenin 2 *				

* detected for the first time in this species.

### 2.2. Total Phenolic Content and Antioxidant Potential of Equisetum Extracts

Oxidative stress describes a pathological state characterized by an imbalance between reactive oxygen/nitrogen species (ROS/RNS) and the body’s antioxidant defenses, resulting in oxidative modification of biological macromolecules such as lipids, proteins and DNA, as well as tissue damage and accelerated cell death. This abnormal oxidative milieu is a fundamental factor in the pathogenesis of many diseases [49].

Therefore, the evaluation of antioxidant activity in biological fluids and foods is valuable in clinical biochemistry for diagnosing and treating diseases related to oxidative stress, comparing antioxidant levels in different foods, and monitoring variations within and between different products.

Natural products contain a variety of antioxidants that have potential therapeutic benefits. These antioxidants work through various mechanisms, such as scavenging primary and secondary radicals, reducing membrane damage caused by free radicals, and sequestering iron to prevent radical formation. Plant polyphenols, which are well-known for their health-promoting properties, exhibit numerous pharmacological effects that are largely due to their antioxidant actions.

The Folin–Ciocalteu (F-C) assay is a useful method for determining the total phenolic content (TPC) as it is easy to use, consistent, and reliable. 

Table 3 shows the results of the determination of total phenolic contents in the five *Equisetum n*-butanol extracts. Chlorogenic acid (CA) and gallic acid (GA) were used as standards. Since CA is a derivative of hydroxycinnamic acid and GA represents a derivative of hydroxybenzoic acid, these two standards represent the phenolic acid derivatives in the samples. It is worth noting that CAE and GAE are commonly used in the literature to assess phenolics completely, including flavonoids, so these assays appeared to be suitable for the present study. Results were expressed as chlorogenic acid equivalents (CAE) and gallic acid equivalents (GAE) per mg dry weight, respectively. The antioxidant capacity values varied significantly, ranging from very low for *E. hyemale* to high for *E. sylvaticum* and *E. telmateia*. The highest phenolic content (Table 3) was found in the extract of *E. telmateia*: 270.9 µg GAE/mg and 514.1 µg CAE/mg, respectively. The lowest phenolic content (Table 3) was found in the extract of *E. hyemale*. Considering the distinct HPLC profiles of the extracts, it is demonstrated that the antioxidant capacity is influenced not only by the total phenolic content but also by the specific phenolic composition. The antioxidant capacity is influenced by the total number of phenolic hydroxyl groups and their position on the aromatic core. The greater the number of ortho- or para-oriented phenolic hydroxyl groups, the higher the antioxidant activity due to the inherent stabilization of the formed radicals via delocalization [50]. It should also be noted that the Folin–Ciocalteu reagent does not only measure the total phenolic content (TPC) but also the total reducing capacity of all compounds in the sample. 

**Figure 5:**
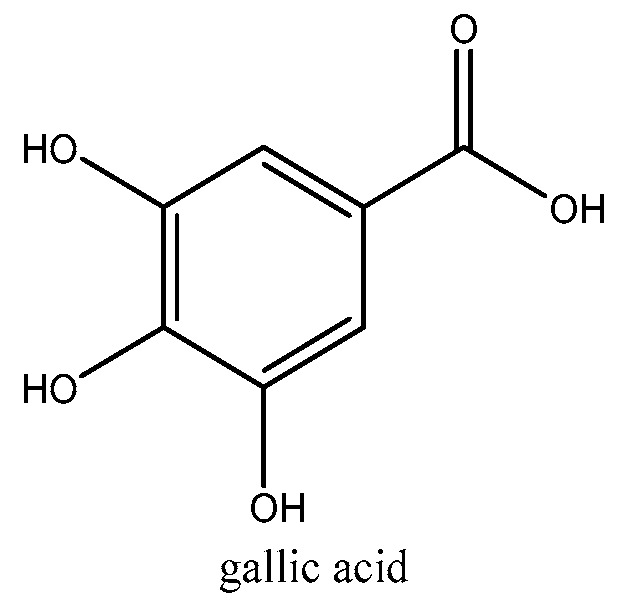
Structure of gallic acid.

**Figure 6:**
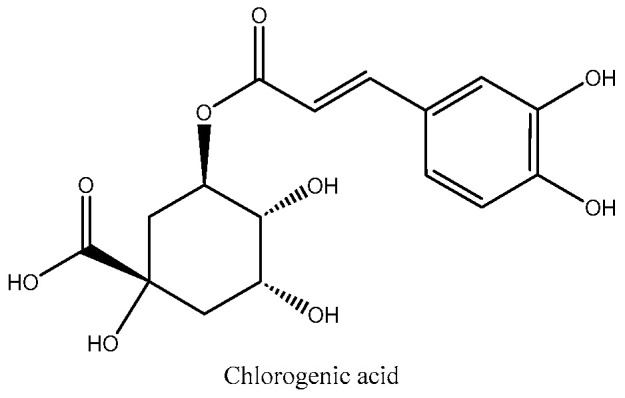
Structure of chlorogenic acid.

The TPC value of chlorogenic acid is almost twice as high as that of gallic acid, indicating that the antioxidant effect of chlorogenic acid is weaker than that of gallic acid. Due to their easily ionizable carboxyl group, phenolic acids are efficient hydrogen donors [51]. The chemical structures of the two phenolic acids used as standards are shown in Figure 5 and Figure 6. Comparing the structures and the antioxidant capacity, the number of hydroxyl groups directly correlates with the antioxidant capacity, which explains why gallic acid is a very strong phenolic antioxidant. Chlorogenic acid has a more complex structure. Two hydroxyl groups are attached to the aromatic group and four others are attached to a saturated cyclohexyl ring. The antioxidant properties of chlorogenic acid are attributed to its unique molecular structure. Specifically, its phenolic hydroxyl structure exhibits high reactivity towards free radicals, producing hydrogen free radicals that have powerful antioxidant effects [51]. 

The same tendency was observed for the radical scavenging activity of the extracts determined by the DPPH assay. The latter is a widely used method for determining the antioxidant activity of various substances and multicompound systems. The DPPH assay was chosen for this study due to its widespread acceptance and reliability in evaluating the free radical scavenging abilities of plant extracts. In this assay, the stable free DPPH radical is reduced by antioxidants, which results in a color change from purple to yellow [52]. The extent of color change is proportional to the antioxidant activity of the tested compounds. Trolox and vitamin C were used as standards and results were expressed as Trolox equivalent antioxidative capacity (TEAC) and vitamin C equivalent antioxidant capacity (VCEAC) per mg dry weight (Table 4). Vitamin C is a natural carbohydrate-like compound with an electron-rich 2-en-2,3-diol-1-one moiety that acts as a strong electron donor. It is predominantly present as an ascorbate anion under physiological pH conditions [53]. Trolox is a synthetic, water-soluble analogue of vitamin E, which is not a natural compound found in foods or plants but a widespread standard for antioxidant measurements [54]. Trolox was used for comparison with lipophilic media, while ascorbic acid was used for comparison with polar compounds. Furthermore, ascorbic acid is common in plant extracts and food and therefore the VCEAC values are of high interest. However, Trolox is more commonly used as a standard in the literature. Thus, it appeared useful to collect both values.

**Figure 7:**
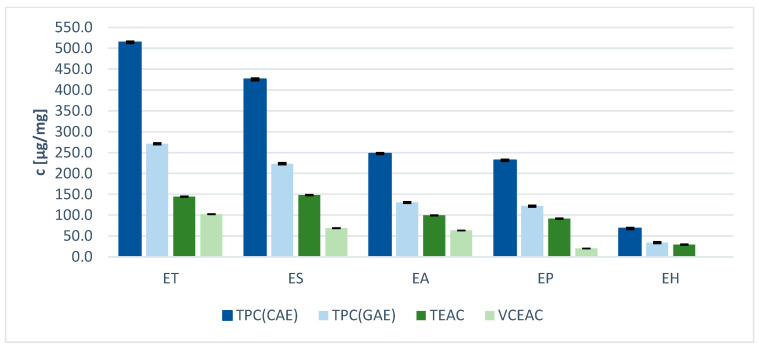
Total phenolic content (TPC) as chlorogenic acid equivalents (CAE) and gallic acid equivalents (GAE) measured using Folin–Ciocalteu assay for 5 extracts from different *Equisetum* species; DPPH based assay as Trolox equivalent antioxidative capacity (TEAC) and vitamin C equivalent antioxidant capacity (VCEAC) for the 5 *Equisetum* species. Bars represent means ± standard deviations (black) (n = 3).

As expected, the order of antioxidant capacity values is almost in line with the TPC results (Figure 7). *E. arvense* showed medium effects, while *E. telmateia* and *E. sylvaticum* exhibited highest activities, and *E. hyemale* displayed lowest. Given the multicompound nature of the extracts, it is challenging to precisely identify which individual compounds are responsible for the antioxidant activity, especially since molecular interaction of compounds in the extract may be synergistic, additive or neutralizing. Table 5 shows the percentage of relative areas under the curve (AUC_rel._) of the UV chromatograms for each *Equisetum* species at 280 nm. The AUC values indicate how the proportions of AUC for flavonoids, AUC for hydroxycinnamic acids (HCA) and AUC for hydroxybenzoic acids (HBA) vary. This demonstrates that the molecular structure of individual compounds is more important than their absolute quantity. As expected, the extract of *E. hyemale*, which contained only few flavonoids, showed a low antioxidant capacity. The large amount of ferulic acid derivatives is responsible for the high percentage of HCA in the area under the curve. The high antioxidant activity of extracts from *E. sylvaticum*, *E. telmateia*, and *E. arvense* may be attributed to their high phenolic contents, including flavonoid glycosides and caffeic acid derivatives. Both flavonoids and caffeic acids contain ortho-oriented phenolic groups. Therefore, it appears that the mono/di-glycosylated and acetylated derivatives have higher antioxidant activities than the tri-glycosylated flavonoid derivatives, such as those found in *E. palustre*. Higher TEAC concentrations occur because it generally takes more Trolox than vitamin C to reduce the same amount of DPPH. This indicates vitamin C to be a stronger antioxidant than Trolox. In summary, these results show that *E. arvense*, *E. telmateia* and *E. sylvaticum* are rich sources of natural antioxidants. 

## 3. Materials and Methods

### 3.1. Chemicals and Reagents

The solvents acetone, acetonitrile, n-butanol (BuOH), dichloromethane (DCM), ethyl acetate (EtOAc), methanol (MeOH), toluene, sodium carbonate and anhydrous sodium sulfate were purchased from Chemsolute (Th. Geyer GmbH & Co., KG, Renningen, Germany). Ascorbic acid and 2,2-diphenyl-1-picrylhydrazyl (DPPH) were from Sigma-Aldrich (St. Louis, MO, USA), and formic acid from Fluka (Sigma Aldrich, St. Louis, MO, USA). Trolox was purchased from Cayman Chemical Company (Ann Arbor, MI, USA), and chlorogenic acid hemihydrate from Alfa Aesar (Karlsruhe, Germany). Folin–Ciocalteu reagent was from Merck KGaA (Darmstadt, Germany). Gallic acid monohydrate was obtained from Carl Roth GmbH & Co. KG (Karlsruhe, Germany).

### 3.2. Plant Material and Extraction

Sterile stems of *Equisetum arvense* were harvested in the medicinal plant garden of WALA Heilmittel GmbH (Bad Boll/Eckwälden, Germany) in August 2022. Sterile stems of *Equisetum telmateia* were collected in Bad Boll/Eckwälden (Germany, 48°37’32.2” N 9°35’41.3” E) in June 2022, *Equisetum hyemale* was collected at the same location as *Equisetum telmateia* in August 2023. Sterile stems of *Equisetum palustre* (48°19’42.8” N 10°00’05.2” E) and *Equisetum sylvaticum* (48°18’42.6” N 9°57’02.7” E) were collected in Ulm, Germany in May 2023. The plant material was subjected to a rigorous cleaning process, followed by coarse fragmentation, after which it was sealed in freezer bags and preserved at a temperature of −70 °C for subsequent investigations. Voucher specimens were deposited at the herbarium of the Institute of Botany, Hohenheim University (Stuttgart, Germany). The identity of the plant material was confirmed by Dr. R. Duque-Thüs (*E. arvense* Bad Boll, voucher number: HOH-022875; *E. telmateia* Bad Boll, voucher number: HOH-022876; *E. hyemale* Bad Boll, voucher number: HOH-022881; *E. palustre* Bad Boll, voucher number: HOH-022882; *E. sylvaticum* Bad Boll, voucher number: HOH-022880).

The extraction process involved 100 g of frozen plant material, which underwent a two-step extraction using a mixture of acetone and water (80/20, *v*/*v*). An amount of 500 mL of solvent was used for each step. To facilitate extraction, the mixture was minced for three minutes using an Ultra-Turrax device at a speed of 15,000 rpm (IKAWerke GmbH and Co., KG, Staufen, Germany). To prevent the oxidative degradation of plant constituents, the mixture was aerated with nitrogen for 10 min before and after mincing.

Afterwards, the resulting mixture was left at a temperature of 4 °C overnight and then filtered using Celite^®^ (Carl Roth GmbH + Co., KG, Karlsruhe, Germany). The solid residue underwent a second extraction as described above. The green-colored filtrates resulting from both extraction steps were combined, and acetone was removed through vacuum rotary evaporation.

Subsequently, the obtained aqueous extract underwent successive extraction with three portions of 150 mL each of dichloromethane, ethyl acetate, and n-butanol using a separating funnel. The dichloromethane and ethyl acetate extracts were dried with anhydrous sodium sulfate and filtered through a glass frit (Por. 3, ROBU^®^ Glasfilter-Geräte GmbH, Hattert, Germany). The solvents were then removed by vacuum rotary evaporation to obtain dry extracts for further analyses. This extraction procedure was carried out twice for all five *Equisetum* species to ensure the reproducibility of the results.

### 3.3. RP-HPLC-DAD-ESI-MS^n^ Analysis

High-performance liquid chromatographic analyses were conducted utilizing an Agilent 1200 HPLC system (Agilent Technologies, Inc., Palo Alto, CA, USA) equipped with a binary pump, micro vacuum degasser, autosampler, thermostatic column compartment, and UV/VIS diode array detector (DAD).

Chromatographic separation was achieved using a Kinetex^®^ C18 reversed-phase column (2.6 µm particle size, 150 mm × 2.1 mm i.d., Phenomenex Ltd., Aschaffenburg, Germany) and a pre-column of the same material at 25 °C with a flow rate of 0.21 mL/min. The mobile phase was 0.1% formic acid in water (eluent A) and acetonitrile (eluent B). Each sample was injected at a volume of 10 µL. The gradient protocol was as follows: 0–10 min, 0–10% B; 10–22 min, 10% B; 22–53 min, 10–23% B; 53–72 min, 23–60% B; 72–80 min, 60–100% B; 80–85 min, 100% B; 85–90 min, 100–0% B; 90–100 min, 0% B.

The LC system was coupled to an HCTultra ion trap mass spectrometer (Bruker Daltonik GmbH, Bremen, Germany) with an ESI source. All extracts underwent analysis in negative ionization mode with a capillary voltage of 4000 V, a dry gas (N_2_) flow of 9.00 L/min, capillary temperature of 365 °C, and nebulizer pressure of 35 psi. 

Full-scan mass spectra (mass range *m/z* 50–1000) of HPLC eluates were recorded during chromatographic separation. MS^n^ data were acquired in the auto MS/MS mode through collision-induced dissociation (CID). Instrument control was managed using ChemStation for LC 3D systems (Rev. B01.03 SR1 (204)) by Agilent and EsquireControl software (V7.1) by Bruker Daltonics. 

Alkaloids were analyzed in the positive ionization mode with the following device parameters: dry gas (N_2_) flow of 8.00 L/min, capillary temperature of 350 °C, and nebulizer pressure of 40 psi. 

BuOH extracts were dissolved in water to achieve a concentration of 4 mg/mL and were passed through a 0.45 µm filter before injection.

### 3.4. Folin–Ciocalteu Assay for the Determination of Total Phenolic Contents

(a) Preparation of calibration standard solutions.

An amount of 470.0 mg gallic acid was weighed into a 10 mL volumetric flask and made up to the mark with water. This stock standard solution had a concentration of c = 47 µg/mL ≙ 2.5 mM.

The calibration standard solutions (c = 1.5–47 µg/mL gallic acid) were prepared by pipetting the indicated amount of stock standard solution and diluting to volume with water. For the chlorogenic acid calibration solutions, the standard was dissolved in water at concentrations from 2.5 to 160 µg/mL.

(b) Preparation of sample test solutions.

An amount of 1.00 mg of accurately weighed plant extract was diluted with deionized water.

(c) Measurement.

Aliquots of 20 µL of sample, water as blank solution and calibration standard solution, respectively, were pipetted in triplicate into a 96-well plate, and 40 µL of Folin–Ciocalteu reagent were added. The plate underwent one minute of shaking in the reader, and subsequently, 160 µL of sodium carbonate solution (700 mM) was added. After an incubation period at 37 °C for 30 min, absorbance at 765 nm was measured using a multiplate reader (Epoch2, Agilent Technologies Inc., Santa Clara, CA, USA).

Total phenolic contents, expressed as gallic acid equivalents [mg gallic acid/mg dry weight], were calculated using linear regression equations.

### 3.5. 2.2-Diphenyl-1-picrylhydrazyl (DPPH) Assay for the Determination of Antioxidant Activity

(a) Preparation of the DPPH solution

An amount of 4.00 mg DPPH was weighed into a 100 mL volumetric flask and made up to the mark with methanol (c = 100 µM).

(b) Preparation of calibration standard solutions.

For the calibration standard solutions, Trolox was dissolved in MeOH at concentrations ranging from 6 to 100 µg/mL, and ascorbic acid was dissolved in MeOH in a concentration range of 2 to 65 µg/mL.

(c) Preparation of sample solutions.

About 1.00 mg of accurately weighed plant extracts were diluted with deionized water.

(d) Measurement.

Aliquots of 30 µL of sample, MeOH as blank solution and calibration standard solution, respectively, were pipetted in triplicate into a 96-well plate and 270 µL of DPPH solution (for blank 270 µL methanol) was added. 

After an incubation period of 45 min at 37 °C, absorbance at 516 nm was measured using a multiplate reader (Epoch2, Agilent Technologies Inc., Santa Clara, CA, USA).

Antioxidant activity, expressed as Trolox equivalent antioxidant capacities (TEAC) [mg Trolox/mg dry weight] and as vitamin C equivalent antioxidant capacities (VCEAC) [mg Vit C/mg dry weight], was calculated using linear regression equations. 

## 4. Conclusions

The present study investigated the polar secondary metabolites and bioactivity characteristics of the sterile stems of five selected *Equisetum* species (Figure 1): *E. arvense* L., *E. telmateia* Ehrh., *E. hyemale* L., *E. sylvaticum* L., and *E. palustre* L. HPLC-DAD-ESI-MS^n^ analyses revealed the chemical diversity within this ancient vascular plant family, comprising hydroxycinnamic acids, hydroxybenzoic acids, flavonoids, and stilbenes. The latter were found for the first time in this plant family. Additionally, our investigation has revealed species-specific chemotaxonomic markers that are useful for differentiating these *Equisetum* species. The absence regarding the trace amounts of caffeic acid derivatives in the UV chromatogram of *E. palustre* is particularly noteworthy, whereas these were present in the other species. Flavonoid tri-glycosides were only present in *E. palustre* and *E. hyemale*, whereas quercetin tri-*O*-hexoside was detected for the first time in these two species. These markers may be useful for quality control of *E. arvense* raw material which is often contaminated with the toxic *E. palustre*. In comparison, *E. hyemale* stood out morphologically and chemically, with ferulic acid derivatives being predominant constituents, while other hydroxycinnamic acid derivatives and flavonoids were only detected in trace amounts. 

The highest phenolic content was determined in the extract of *E. telmateia* (270.9 µg GAE/mg Ex and 514.1 µg CAE/mg Ex), lowest were determined in the extract of *E. hyemale* (34.2 µg GAE/mg Ex and 68.3 µg CAE/mg Ex). *Equisetum sylvaticum* exhibited the highest DPPH radical scavenging activity, while *Equisetum arvense* showed moderate activity and *Equisetum hyemale* showed the lowest activity. The identified compounds, including hydroxycinnamic acids, hydroxybenzoic acids, flavonoids, and stilbenes, have been associated with various bioactivities in previous studies. For instance, flavonoids and hydroxycinnamic acids are known for their antioxidant properties, which were confirmed in this study. Future research shall include detailed bioassays to evaluate antifungal, antibacterial, and anticancer activities, enabling a comprehensive understanding of how these phytochemicals contribute to the pharmacological properties of *Equisetum* species.

This comprehensive analysis contributes valuable insights into the chemical diversity and pharmacological potential of the aforementioned *Equisetum* species. Furthermore, this study enhances our understanding of how phytochemical composition varies within the Equisetaceae family. The practical applications of these findings are significant, particularly in the fields of herbal medicine. The identification of specific chemotaxonomic markers can improve the accuracy and safety of *Equisetum*-based products, ensuring that only non-toxic species are used in traditional and commercial preparations. Furthermore, the discovery of unique compounds opens new avenues for the development of natural agents, which could be utilized in dietary supplements, cosmetics, and pharmaceuticals. Future research should focus on a more detailed exploration of the bioactivities of individual compounds identified in this study. Investigations into the antifungal, antibacterial, and anticancer properties of these compounds, as well as their mechanisms of action, would provide a deeper understanding of their therapeutic potential. Additionally, studies on the synergistic effects of these compounds could further elucidate their roles in the overall bioactivity of *Equisetum* extracts. 

In conclusion, this comprehensive analysis not only enhances our understanding of the chemical diversity and pharmacological potential of *Equisetum* species but also provides valuable insights for their practical applications in various industries. It is anticipated that further research in this field will result in the development of novel, natural therapeutic agents derived from these ancient plants. 

## Figures and Tables

**Table 3:** Total phenolic content (TPC) determined by Folin–Ciocalteu method in various extracts (Ex) from *Equisetum* species in descending order. Data are presented as mean ± standard deviation (n = 3) as chlorogenic acid equivalents (CAE) and gallic acid equivalents (GAE).

Sample	TPC [µg GAE/mg Ex]	TPC µg [CAE/mg Ex]
*Equisetum telmateia*	270.9 ± 0.50	514.1 ± 0.94
*Equisetum sylvaticum*	223.4 ± 0.69	425.5 ± 1.28
*Equisetum arvense*	130.4 ± 0.19	247.6 ± 0.36
*Equisetum palustre*	121.8 ± 0.34	231.5 ± 0.64
*Equisetum hyemale*	34.2 ± 0.44	68.3 ± 0.81

**Table 4:** Antioxidant activity assessed by DPPH method in various extracts (Ex) from *Equisetum* species in descending order. Data are presented as mean ± standard deviation (n = 3) as Trolox equivalent antioxidative capacity (TEAC) and vitamin C equivalent antioxidant capacity (VCEAC).

Sample	VCEAC [µg Vit C/mg Ex]	TEAC [µg Trolox/mg Ex]
*Equisetum sylvaticum*	102.5 ± 0.42	148.3 ± 0.61
*Equisetum telmateia*	99.8 ± 0.35	144.4 ± 0.51
*Equisetum arvense*	68.9 ± 0.24	99.6 ± 0.35
*Equisetum palustre*	63.5 ± 0.18	91.8 ± 0.27
*Equisetum hyemale*	20.5 ± 0.32	29.5 ± 0.47

**Table 5:** The percentage of relative area under the curves (AUC) in RP-HPLC-DAD-MS^n^ UV chromatograms at 280 nm (s. Table 1) for flavonoids, hydroxycinnamic acids (HCA), and hydroxybenzoic acids (HBA).

Sample	${AUC}_{rel.}\left(Flavonoid\right)$ [%]	${AUC}_{rel.}\left(HCA\right)$ [%]	${AUC}_{rel.}\left(HBA\right)$ [%]	$\sum {AUC}_{rel.}$ [%]
*Equisetum telmateia*	38.6	21.9	0.1	60.6
*Equisetum sylvaticum*	29.9	38.7	1.1	69.7
*Equisetum arvense*	46.2	42.6	0.0	88.8
*Equisetum palustre*	64.3	20.4	0.1	84.8
*Equisetum hyemale*	2.3	88.9	0.3	91.6

## Data Availability

The original contributions presented in the study are included in the article, further inquiries can be directed to the corresponding author.
